# School-Based Rescued Produce Distribution, and Changes in Parental Fruit Intake and Household Dietary Behaviors: A Non-Randomized Controlled Cluster Trial

**DOI:** 10.3390/nu18203355

**Published:** 2026-10-13

**Authors:** Smadar Hod-Ovadia, Moran Kilzi, Hagai Factor, Mona Boaz, Vered Kaufman-Shriqui

**Affiliations:** 1 Department of Nutrition Sciences, Faculty of Health Sciences, Ariel University, Ariel 40700, Israel; smadar@leket.org (S.H.-O.); moran@leket.org (M.K.); monabo@ariel.ac.il (M.B.); 2 Leket Israel, Ra’anana 43650, Israel; 3 Department of Psychology, Faculty of Humanities and Social Sciences, Ariel University, Ariel 40700, Israel; hagaifactor@gmail.com; 4 The Center for Urban Health Solutions (C-UHS), St. Michael’s Hospital, Toronto, ON M5B 1T8, Canada

**Keywords:** food security, fruit and vegetable intake, school-based intervention, food rescue, produce distribution, parental dietary behavior, nutrition education, nutrition equity

## Abstract

**Background/Objectives:** Fruit and vegetable intake remains below recommended levels, particularly among families facing socioeconomic constraints. School-based food access interventions may improve household dietary behaviors. This study evaluated the association between participation in the “Leket Health” school-based rescued produce distribution program and changes in parental fruit and vegetable intake, nutrition-related parenting practices, and household food security. Children’s fruit and vegetables intake was assessed as a secondary exploratory outcome. **Methods**: A non-randomized controlled cluster trial was conducted among parents (*n* = 281) of 4th–5th-grade students at three elementary schools in a peripheral town in Israel, with one school per study arm (three clusters in total). Schools were assigned to full intervention (biweekly fruit and vegetable boxes and nutrition education), partial intervention (boxes only), or control. Parent data were collected at baseline and after 7 months using both self-reported fruit and vegetable portions and a validated food frequency questionnaire (FFQ), food security questionnaire, and parental behavior questionnaire. Child intake was assessed using a study-specific FFQ. Primary analyses used a complete-case approach, supplemented by an allocation-based multiple-imputation sensitivity analysis among 269 identifiable participants. The trial was not registered in a public registry. **Results**: A total of 70% of enrolled participants completed the study. The two parental intake measures yielded discordant findings: categorical changes in parent-reported daily fruit servings differed across study groups in the complete-case analysis (*p* = 0.028), whereas FFQ-derived fruit and vegetable intake (g/day) showed no statistically significant between-group differences. The overall parenting-practices score showed no statistically significant change (*p* = 0.770) or between-group difference (*p* = 0.297). Household food insecurity increased from 8.7% to 14.3% and worsened across all study arms, with no significant between-group difference in deterioration (*p* = 0.810). No primary dietary endpoint was prespecified. In the allocation-based multiple-imputation analysis, adjusted follow-up fruit intake was higher than control by 0.38 portions/day (95% CI 0.04–0.71) in the full-intervention arm and 0.25 portions/day (95% CI −0.10 to 0.61) in the partial-intervention arm; estimates were attenuated under more adverse assumptions regarding missing outcomes. In an exploratory post-allocation adherence analysis, each additional produce box collected was associated with 6.7% higher odds of a parent-reported increase in fruit intake (OR = 1.067, 95% CI 1.003–1.135; *p* = 0.040). Among intervention participants, parent-reported improvements in nutrition-related parenting practices were associated with parent-reported increases in household vegetable variety (OR = 2.219, 95% CI 1.057–4.656; *p* = 0.035). These exploratory associations do not establish intervention effects. The overall parenting-practices score showed no statistically significant change (*p* = 0.770) or between-group difference (*p* = 0.297). Household food insecurity increased from 8.7% to 14.3% and worsened across all study arms, with no significant between-group difference in deterioration (*p* = 0.810). Among children, no significant changes were observed in FFQ-derived intake. **Conclusions**: Program participation was associated with higher parent-reported fruit servings, although the magnitude of between-arm differences varied across missing-data assumptions. No significant changes were observed in parental vegetable servings or FFQ-derived fruit and vegetable intake among parents or children. Given the single-school-per-arm design, these findings should be interpreted as exploratory and require confirmation in studies with multiple schools per arm.

## 1. Introduction

Fruit and vegetable consumption constitutes a fundamental component of a healthy dietary pattern and is consistently associated with reduced risk of chronic diseases, including cardiovascular disease, some cancers, and overall mortality [1,2,3,4]. National dietary guidelines, including those in Israel, recommend consuming at least 5 servings of vegetables and 3 servings of fruit daily [5]. Despite these recommendations, adherence remains suboptimal across populations, particularly among individuals and families of lower socioeconomic status in high-income countries, including Israel [6,7,8,9].

Recent national surveys in Israel demonstrate a concerning decline in fruit and vegetable consumption over time, with only a minority of adults meeting recommended intake levels [10,11]. This gap reflects a complex interplay of individual, environmental, and socioeconomic determinants. Key barriers include cost, limited access, lack of time, insufficient knowledge, and competing household priorities [12,13].

Food insecurity represents a critical determinant of dietary quality and refers to limited or uncertain access to sufficient, safe, and nutritious food that meets dietary needs and food preferences for an active and healthy life [14,15]. Evidence suggests that food insecurity is strongly associated with lower intake of fruits and vegetables and higher consumption of ultra-processed foods [16,17]. These dietary patterns contribute to the burden of chronic diseases, including obesity, diabetes, and cardiovascular conditions [2,3,18,19]. Therefore, interventions that improve access to fruits and vegetables and support their integration into daily household routines may contribute to both improved diet quality and the reduction of nutrition-related health inequalities.

Interventions designed to address these disparities have included financial incentives, food vouchers, produce prescription programs, and education-based approaches. Programs offering financial incentives have demonstrated modest improvements in fruit and vegetable intake, whereas combined interventions integrating food provision with education may be more effective in supporting dietary behavior change [20,21,22,23].

Schools have increasingly been recognized as effective platforms for implementing nutrition interventions, given their accessibility and ability to reach both children and families. While most school-based interventions focus primarily on children, emerging evidence suggests that such programs may also influence parental behaviors, particularly when combined with family engagement strategies and changes in the home food environment [21,24,25,26].

The “Leket Health” program represents a novel school-based public health nutrition approach that integrates the distribution of rescued agricultural produce with nutrition education. The program provides biweekly boxes of fresh fruits and vegetables to families of elementary school students and includes educational activities designed to increase awareness, acceptability, and use of fruits and vegetables. Importantly, the program provides produce boxes universally to families within participating schools, thereby minimizing stigma, increasing accessibility, and promoting inclusivity.

The present study examined differences among three schools in the “Leket Health” program on parental dietary behaviors, including fruit and vegetable consumption and nutrition-related parenting practices, and examined the role of food security in moderating these outcomes. Given the limited evidence on parental outcomes of school-based produce distribution programs, this study also sought to contribute to the understanding of how non-stigmatizing food access interventions may support household dietary behaviors in socioeconomically vulnerable communities. Child-reported FFQ-derived fruit and vegetable intake is also reported as a secondary exploratory measure. No directional hypothesis for these contrasts or primary dietary endpoint was prespecified.

## 2. Materials and Methods

### 2.1. Study Design and Setting

A non-randomized cluster-controlled trial was conducted in three elementary schools in a peripheral town in Israel, during the 2023–2024 school year.

### 2.2. Participants

All elementary schools in the city were approached through the municipal Education Department and invited to participate. Schools were eligible if they enrolled students in the relevant age and grade groups and if the school principal expressed willingness to participate in the program. The study included three schools, with one school per study arm. Schools were selected for the intervention arms based on their principals’ willingness to participate in the program; allocation was not randomized. Once eligible schools had been identified, the project was presented to all parents or primary caregivers of students in the selected 4th–5th-grade classes, who were invited to enroll themselves and their children in the study. The study included three schools, with one school per study arm. Schools were selected for the intervention arms based on their principals’ willingness to participate in the program; allocation was not randomized.

Eligible participants in the broader study included 4th–5th-grade students attending the participating schools and one parent or primary caregiver per participating student. For the present article, parent-reported data were used for the primary outcomes, while child-reported FFQ-derived fruit and vegetable intake was analyzed as a secondary exploratory measure.

Parent participants were eligible if they were a parent or first-degree caregiver of a participating student, were able to complete the study questionnaires in Hebrew or Russian, and provided informed consent. Only one parent or primary caregiver per child was eligible to participate. Children were eligible if they were enrolled in grades 4–5 at a participating school, could complete the child questionnaires in Hebrew, and had parental consent to participate.

Exclusion criteria for parents included inability to complete the questionnaire in either study language or affiliation with school staff when the participant’s child attended a school assigned to a different study arm. Children who were not enrolled in grades 4–5, did not attend one of the participating schools, or were unable to complete the child questionnaires in Hebrew were excluded.

### 2.3. Intervention

The intervention was implemented over a 7-month period and included three study arms: (1) Full intervention, consisting of biweekly distribution of fruit and vegetable boxes each weighing approximately 12 kg, combined with nutrition education activities; (2) Partial intervention, consisting of produce box distribution only; And (3) control group, with no intervention during the study period.

A total of 15 produce distributions were conducted in the two intervention schools. The produce boxes included a variety of fresh fruits and vegetables sourced from rescued agricultural produce available through Leket Israel. On average, fruit accounted for approximately 37% of the box contents.

In the full intervention group, each produce box also included an educational information sheet and a recipe tailored to the specific produce in that box. The nutrition education component included 4 classroom-based sessions for students and 2 planned parent-directed sessions. The children’s lessons introduced a variety of fruits and vegetables and their nutritional properties and health benefits, incorporated tasting activities and the preparation of simple recipes, lasted approximately 60 min each, and were delivered by a registered dietitian. The parent session focused on fruit and vegetables, combining factual information with practical strategies to encourage their consumption among children and adults. It lasted approximately 60 min and was delivered by a registered dietitian. This component was designed to promote awareness of, acceptability of, storage, preparation, and practical use of fruits and vegetables among students and families, and is part of the structured lessons and materials developed specifically for schools by the Leket Health program.

### 2.4. Data Collection and Measures

Parents completed self-administered online questionnaires at baseline and post-intervention. Questionnaires were developed using Qualtrics and distributed via electronic links. The questionnaires were available in Hebrew and Russian, which are the common languages among residents of this area. Children completed structured computerized questionnaires during school hours in school computer classrooms at two time points: baseline and after 7 months of program implementation.

The survey package included demographic and socioeconomic measures adapted from Israeli national surveys [7,27,28]. These included age, sex, country of birth, immigration date, years of education, employment status, household size, number of children under 18 years of age, ability to cover monthly household expenses, and self-reported household income relative to the national average. Child demographic variables included sex, grade, and country of birth.

Household food security was assessed with the validated 18-item USDA-based food security questionnaire adapted for households with children [27,29]. Responses were used to classify households into four categories: high, marginal, low, and very low food security.

Parental fruit and vegetable intake was assessed using two measures: a validated Israeli food frequency questionnaire comprising 116 food items [30] and by self-reported usual daily servings, with serving sizes defined according to the Israeli Ministry of Health food exchange guidelines [31]. Change in fruit and vegetable intake was calculated as the difference between the number of servings reported at follow-up and at baseline and was categorized as decreased, unchanged, or increased. For parents reporting low intake, additional study-developed items assessed perceived reasons for consuming fewer than three daily servings of vegetables or fewer than two daily servings of fruit.

Child-reported fruit and vegetable intake was assessed using a study-specific FFQ focused exclusively on these foods. The questionnaire was developed for the present study, adapted from an existing child FFQ [32] and included age-appropriate images of commonly consumed fruits and vegetables to support reporting. For the present article, child FFQ-derived outcomes included estimated daily fruit and vegetable intake (in grams) and were analyzed as secondary exploratory measures.

Nutrition-related parenting practices were assessed using items addressing food preparation, serving practices, and the provision of fruits and vegetables to children [24,33]. These measures included parental actions promoting children’s fruit and vegetable consumption, such as incorporating fruits and vegetables into meals, offering them between meals, preparing vegetable-based dishes, and using fresh produce in home cooking.

At follow-up, additional items assessed reported use of the produce boxes among parents in the intervention groups. Items addressed family consumption of the fruits and vegetables received, and the variety of fruits and vegetables consumed. Responses were rated on a four-point scale: usually true, sometimes true, not true, and do not know.

### 2.5. Sample Size

Sample size was calculated using G*Power software (version 3.1.9.7 for Windows; Heinrich Heine University Düsseldorf, Düsseldorf, Germany). The calculation aimed to detect a difference of 0.5 fruit and vegetable servings in parental intake, assuming a standard deviation of 1 serving, a significance level of α = 0.05, and 80% power. Based on these assumptions, the target sample size was 60 parents per study group. Child-reported FFQ-derived fruit and vegetable intake was analyzed as a secondary exploratory outcome and was not the basis for the primary sample size calculation. The G*Power calculation was based on independent individual observations and did not account for intracluster correlation or the number of participating schools. It therefore provided an individual-level recruitment target rather than a sample-size justification for a cluster trial.

### 2.6. Statistical Analysis

Data were analyzed using SPSS version 29. Continuous variables were examined for normality using the Kolmogorov–Smirnov test and were presented as mean ± SD or median (interquartile range), as appropriate. Categorical variables are presented as frequencies and percentages. Between-group comparisons were conducted using one-way ANOVA or Kruskal–Wallis tests for continuous or ordinal variables and chi-square or Fisher’s exact tests for categorical variables, with Bonferroni correction for multiple comparisons when appropriate. Child demographic characteristics were summarized by study group using frequencies and percentages. Child-reported FFQ-derived fruit and vegetable intake is presented as mean ± SD. Paired baseline-to-follow-up comparisons used paired tests, and between-group comparisons of change used one-way ANOVA on individual change scores. With one school per arm, school identity was completely confounded with intervention condition, so individual-level comparisons were considered exploratory. Binary logistic regression models were fitted to examine exploratory associations with increased fruit intake and increased household vegetable variety. Increased fruit intake was defined as any positive change in self-reported daily fruit servings from baseline to follow-up; unchanged or decreased intake formed the reference category. Increased vegetable variety was defined as the number of items endorsed on the corresponding item in the follow-up questionnaire. Associations are reported as odds ratios (ORs) with 95% confidence intervals (CIs). The produce-box model was a post-allocation adherence analysis and was adjusted for reported ability to cover monthly household expenses; it was not interpreted as an analysis by original allocation.

Primary analyses followed a complete-case approach and included participants with the relevant measurements available at both baseline and follow-up. For multivariable analyses, complete data for the variables included in each model were also required. Consequently, analytic sample sizes varied by outcome and model. Missing values were not imputed for these primary analyses.

Retention in the final analytic sample was summarized by the originally allocated study arm and compared using an exploratory Pearson chi-square test. Participants without a completed baseline assessment, those without follow-up, and those excluded for other reasons were distinguished in the participant flow diagram.

To assess the robustness of the complete-case findings to missing data, an allocation-based sensitivity analysis was conducted among all 269 identifiable enrolled participants, who were retained in their originally assigned school-level study arms. Missing baseline and follow-up fruit-intake values and covariates were multiply imputed within study arm using 100 imputations under a missing-at-random assumption. The imputation model included baseline and follow-up parent-reported fruit servings/day, parental age (years), education (years), and baseline household income code. Income code 4 was treated as missing; imputed fruit servings were rounded to whole numbers and constrained to 1–8, and imputed income codes were constrained to 1–3. Adjusted follow-up fruit-intake means were then standardized for baseline fruit intake, parental age, education, and baseline household income category.

Two additional sensitivity scenarios were examined to assess the influence of alternative assumptions regarding missing follow-up outcomes. First, missing follow-up fruit intake was set equal to the participant’s observed or imputed baseline value, representing no change in intake. Second, a more conservative scenario was examined in which one portion/day was subtracted from imputed follow-up values for intervention participants with missing follow-up outcomes, with values constrained to a minimum of one portion/day. Between-arm contrasts from these analyses were interpreted descriptively because each study arm comprised a single school, and school effects could not be separated from intervention effects. All tests were two-sided, and *p* < 0.05 was considered statistically significant.

### 2.7. Ethics

The trial received ethics approval from the IRB committee of the Israeli Ministry of Education (Protocol number 12769) and from Ariel University (ethics approval number AU-HEA-VKS-20240122) for the same protocol.

Written informed consent was obtained from all participating parents prior to enrollment. Parental consent also covered children’s participation in the school-based questionnaires, and children were informed that participation was voluntary and that they could decline to answer. The trial was not registered in a public trial registry. Reporting of the school-level design was informed by relevant items from the CONSORT extension for cluster-randomized trials [33], adapted to the non-randomized design of this study. The adapted reporting checklist is provided in Supplementary Table S1.

## 3. Results

### 3.1. Participant Recruitment and Retention

**Figure 1:**
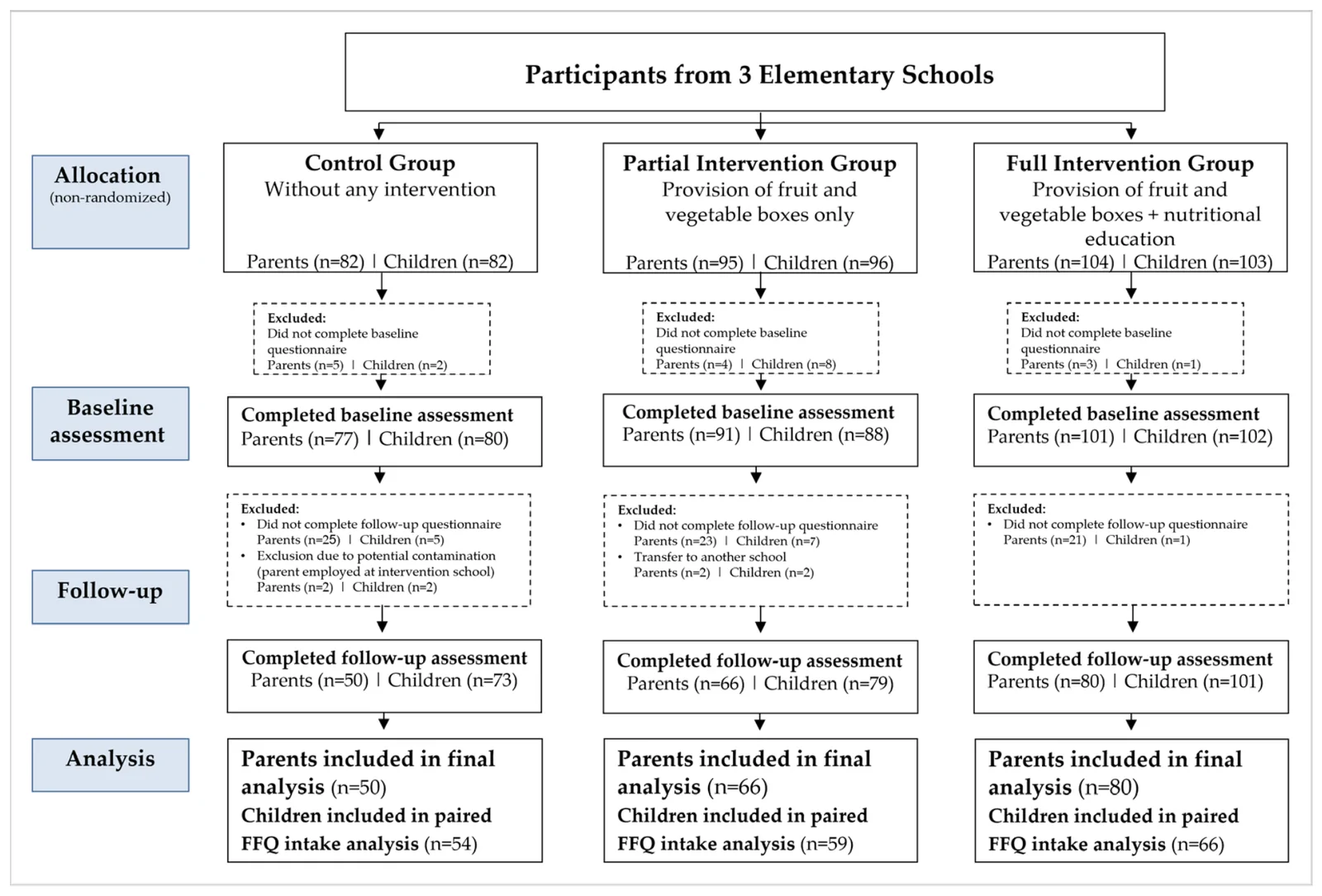
Study flow diagram. The diagram presents participant allocation, baseline assessment, follow-up, and final analysis across the three study groups.

Participant recruitment, retention, and analytic samples across study groups are presented in Figure 1. A total of 281 parents and 281 children provided informed consent and were enrolled in the study, representing an overall response rate of 81%. Of the 281 enrolled parents, 196 were included in the final analysis. The remaining 85 comprised 12 without a completed baseline assessment, 69 without follow-up, and 4 excluded for other reasons. Retention in the final analytic sample was 80/104 (76.9%) in the full intervention arm, 66/95 (69.5%) in the partial intervention arm, and 50/82 (61.0%) in the control arm (nominal Pearson χ^2^(2) = 5.532, *p* = 0.063). Participants lost to follow-up had significantly lower educational attainment than those retained at follow-up (13.46 ± 2.62 vs. 14.79 ± 3.45 years of education, respectively; *p* < 0.001). Of these, 196 parents completed both baseline and follow-up questionnaires and were included in the final analysis, corresponding to an approximately 70% completion rate. Among children, 253 completed the follow-up assessment, and 179 children with paired FFQ data were included in the secondary exploratory analysis of child-reported fruit and vegetable intake.

### 3.2. Baseline Characteristics

Baseline demographic and socioeconomic characteristics of parent, household, and child participants are presented in Table 1. The parent analytic sample included 196 parents, most of whom were mothers (88.8%), with a mean age of 40.7 ± 5.1 years. The mean household size was 4.9 ± 1.2 persons, with 2.7 ± 1.1 children under 18 years of age per household. Most households reported high food security at baseline. No statistically significant baseline differences were observed between study groups for parent or household characteristics. Among children, the distribution of sex, grade, and country of birth was generally comparable across study groups. No statistically significant baseline differences were observed between study groups for the child characteristics examined.

### 3.3. Baseline Parental Fruit and Vegetable Intake

At baseline, average intake estimated from the food frequency questionnaire was 410.6 ± 587.6 g/day for vegetables and 256.1 ± 345.4 g/day for fruits, with no significant baseline between-group differences (vegetables: *p* = 0.417; fruits: *p* = 0.423).

**Figure 2:**
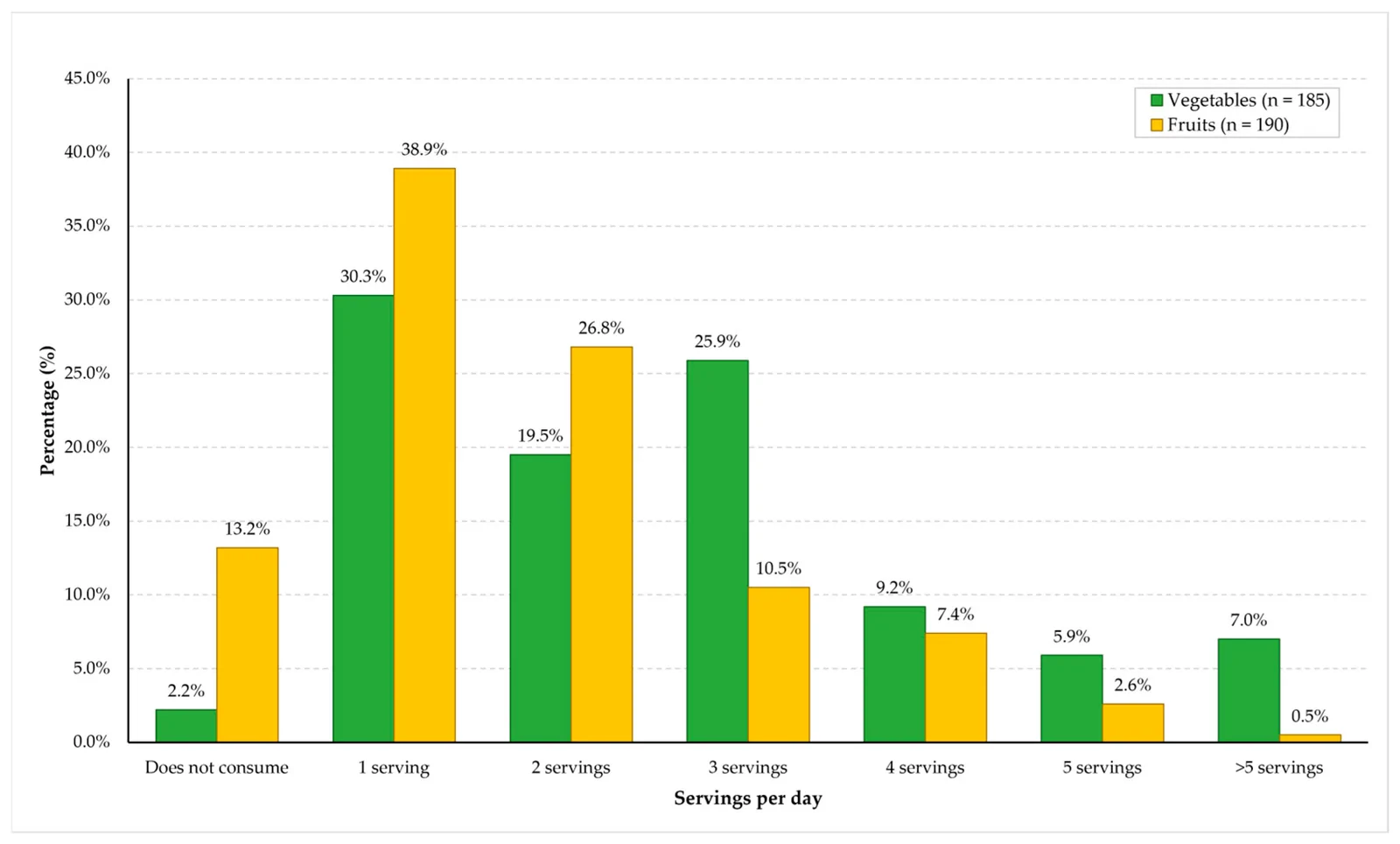
Baseline parental daily fruit and vegetable intake. This figure presents the distribution of parents’ daily fruit and vegetable intake by number of servings consumed at baseline among study participants. Values are presented as percentages of respondents within each intake category.

**Figure 3:**
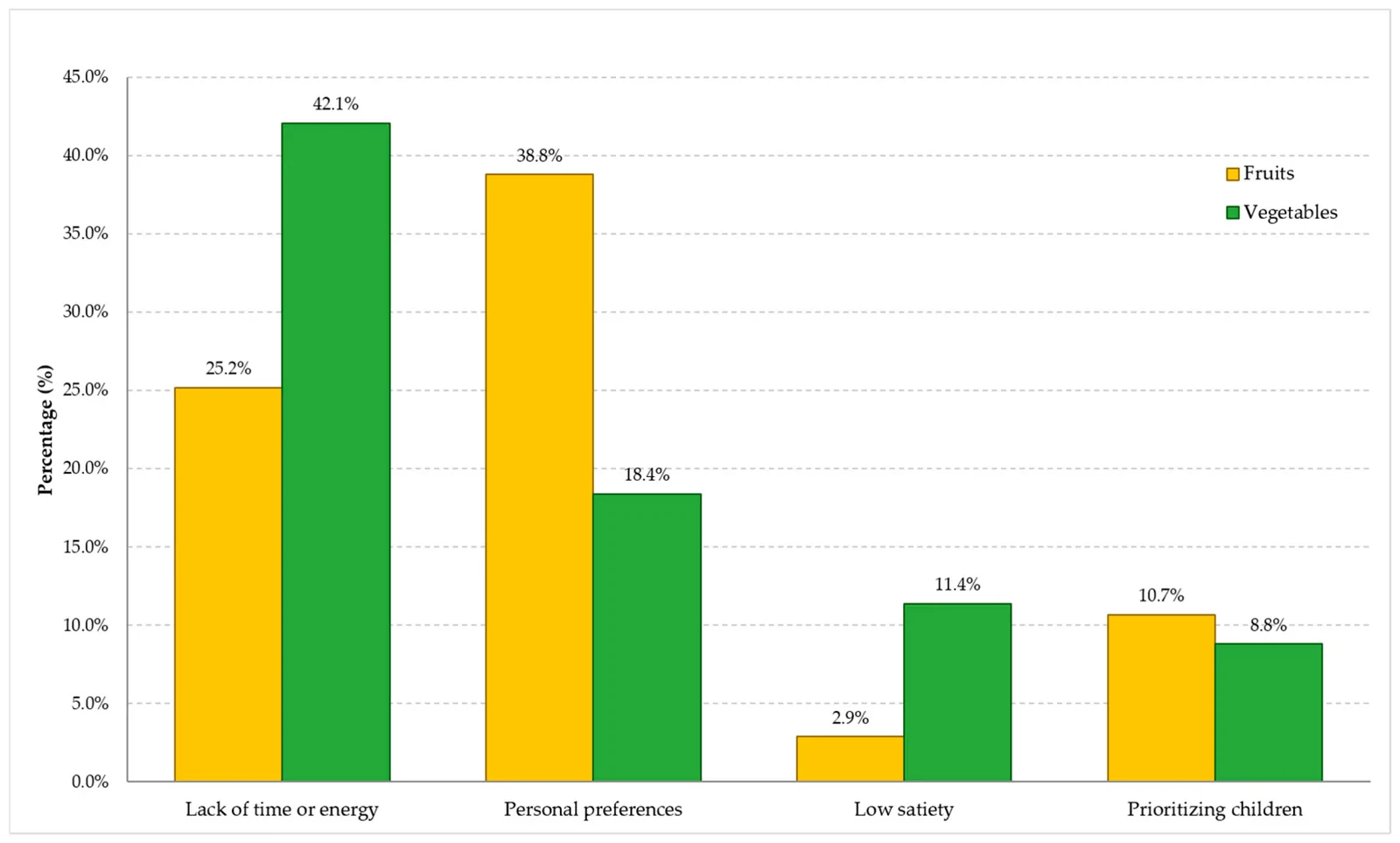
Parent-reported barriers to fruit and vegetable consumption at baseline. The figure presents the distribution of primary self-reported barriers to adequate fruit and vegetable consumption among study participants at baseline. Values are presented as percentages. Reported reasons included lack of time or energy, personal preferences, low satiety, and prioritization of children’s consumption.

In a separate question assessing usual daily servings, with serving sizes defined according to the Israeli Ministry of Health food exchange guidelines, self-reported daily servings indicated low intake among most parent participants. Overall, 77.9% of parents reported consuming fewer than three daily servings of vegetables, and 78.9% reported consuming fewer than two daily servings of fruit. The distribution of daily parental fruit and vegetable servings at baseline is presented in Figure 2. The most frequently reported reason for low intake was lack of time or energy to handle these foods. As shown in Figure 3, this barrier was reported more frequently for vegetables than for fruits. Other commonly reported barriers included personal preferences, low satiety, and prioritizing children’s consumption over the parents’ own intake.

### 3.4. Intervention Exposure and Use of Produce Boxes

During the intervention period, 15 biweekly fruit and vegetable box distributions were conducted in the full and partial intervention groups. Adherence was significantly higher in the full intervention group: 60 participants (76%) collected 13 or more boxes, compared with 26 participants (39%) in the partial intervention group. The mean number of boxes collected was also higher in the full intervention group than in the partial intervention group (12.71 ± 3.17 vs. 9.21 ± 5.13 boxes, respectively; *p* < 0.001).

At follow-up, additional items assessed reported use of the produce boxes among parents in the intervention groups. Overall, household use of the produce boxes was high. Most parents reported that their families consumed most of the fruit (92.4%) and vegetables (91.9%) received in the boxes, with no significant differences between the full- and partial-intervention groups. In addition, 63.6% reported improved fruit variety and 63.9% reported improved vegetable variety in the household.

In the full intervention group, the student-directed nutrition education component was implemented as planned, with four classroom-based sessions delivered during the intervention period. The family-directed educational component was implemented by including an information sheet and a recipe in each produce box, tailored to the specific produce. Following the outbreak of the Israel–Hamas war in October 2023, the planned parent-directed group education component was modified. One online lecture was delivered to parents in the full intervention group, with 12 of 104 invited parents participating (11.5%).

### 3.5. Changes in Parental Fruit and Vegetable Consumption

In the complete-case analysis, parent-reported increases in daily fruit servings were more frequent in the full-intervention (28.0%) and partial-intervention (32.8%) schools than in the control school (14.3%), with a between-group difference in categorical change (*p* = 0.028), whereas FFQ-derived fruit and vegetable intake (g/day) showed no statistically significant between-group differences. The corresponding descriptive differences versus the control school were 13.7 and 18.5 percentage points, respectively (Table 2). No statistically significant between-group difference was observed for categorical changes in parent-reported vegetable servings (*p* = 0.738).

Changes in parental fruit and vegetable consumption following the intervention are presented in Table 2. Analysis of parental FFQ data showed small, non-significant changes in average fruit and vegetable intake in grams/day from baseline to follow-up, with no significant between-group differences.

Three allocation-based sensitivity analyses were conducted among the 269 identifiable participants retained in their originally assigned school-level arms: multiple imputation under MAR, missing follow-up equal to baseline (no-change), and adverse missingness (Table 3; Supplementary Table S2). Under MAR, adjusted mean follow-up parent-reported fruit intake was 2.81 (95% CI 2.58–3.04), 2.69 (95% CI 2.45–2.92), and 2.44 (95% CI 2.18–2.69) portions/day in the full-intervention, partial-intervention, and control arms, respectively. Adjusted differences versus control under all three assumptions are presented together in Table 3.

Compared with MAR, the no-change assumption reduced the full-arm contrast from +0.38 to +0.29 portions/day, while the partial-arm contrast was similar (+0.25 versus +0.27). Both contrasts were smaller under adverse missingness (+0.15 and +0.06 portions/day, respectively). These sensitivity analyses assess dependence on missing-data assumptions and do not provide independent confirmation of a dietary change. Confidence intervals are nominal individual-level estimates conditional on the participating schools and cannot separate school-level differences from intervention effects.

### 3.6. Post-Allocation Adherence Analysis of Parent-Reported Increases in Fruit Servings

An exploratory post-allocation logistic regression analysis was conducted to identify factors associated with increased fruit consumption (Table 4). Each additional produce box collected was associated with a 6.7% increase in the odds of improved fruit intake (OR = 1.067, 95% CI 1.003–1.135, *p* = 0.040), independent of household socioeconomic status. This analysis reflects post-allocation intervention exposure and should not be interpreted as an analysis by original study allocation.

### 3.7. Child-Reported FFQ-Derived Fruit and Vegetable Intake

Child-reported FFQ-derived fruit and vegetable intake was examined as a secondary exploratory outcome among children with paired baseline and follow-up FFQ data (*n* = 179). Mean fruit intake showed a small, non-significant increase from 433 ± 289 g/day at baseline to 437 ± 320 g/day at follow-up (*p* = 0.450). Mean vegetable intake decreased non-significantly from 325 ± 184 g/day at baseline to 294 ± 172 g/day at follow-up (*p* = 0.062). No statistically significant between-group differences were observed for either fruit intake (*p* = 0.997) or vegetable intake (*p* = 0.843).

### 3.8. Changes in Household Food Security

**Figure 4:**
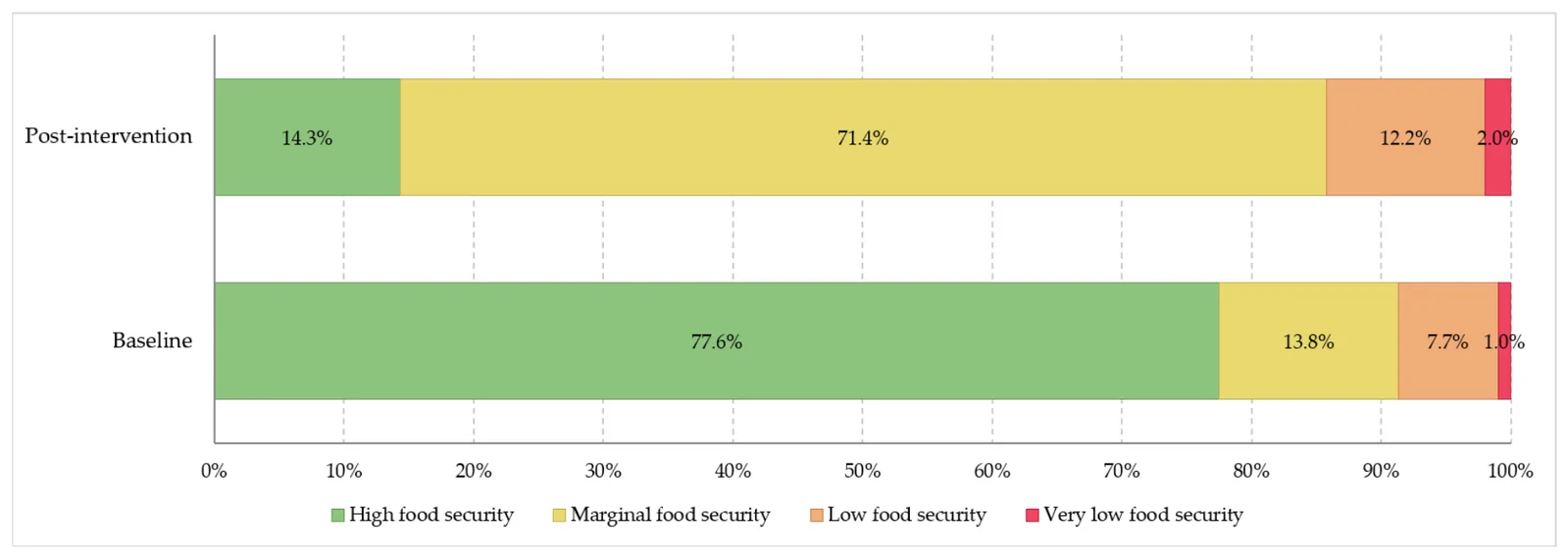
Changes in household food security status from baseline to post-intervention. Food security is classified into four categories based on validated questionnaire scoring. Changes reflect shifts in household food security status during the study period.

Household food security deteriorated over the study period (Figure 4). The proportion of households classified as food insecure (low or very low food security) increased from 8.7% at baseline to 14.3% at follow-up. The overall food security score worsened significantly from baseline to follow-up (t(195) = 11.021, *p* < 0.001). However, the degree of deterioration in food security did not differ significantly between the study groups (*p* = 0.810).

### 3.9. Association Between Household Food Security Deterioration and Parental Fruit Intake

An exploratory two-way ANOVA examined the association between change in parent-reported household food security status, study group, and change in parental fruit intake. Among parents whose household food security deteriorated, fruit intake was 0.23 servings higher in the full intervention group than in the control group, with a significant interaction between study group assignment and household food security deterioration (F(1,113) = 7.686, *p* = 0.007, partial η^2^ = 0.064).

### 3.10. Changes in Nutrition-Related Parenting Practices

**Figure 5:**
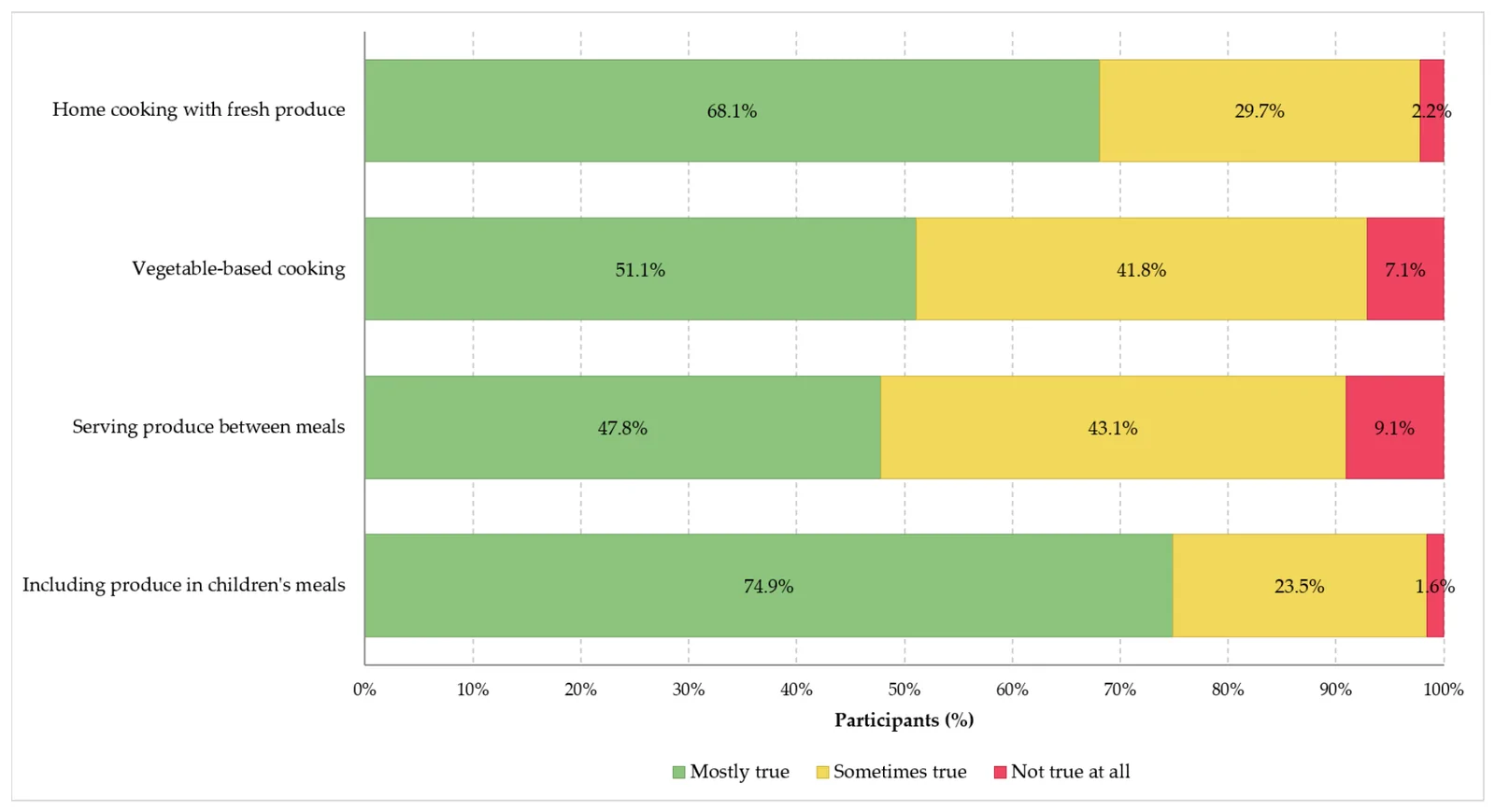
Parental practices to promote children’s fruit and vegetable consumption at post-intervention. The figure presents the distribution of parental responses regarding practices that support children’s fruit and vegetable consumption at post-intervention. Values are presented as percentages across three response categories: mostly true, sometimes true, and not true at all.

Figure 5 presents the distribution of nutrition-related parenting practices at follow-up. Nutrition-related parenting practices improved modestly from baseline to follow-up. The proportion of parents who reported usually including fruits or vegetables in meals prepared for their children increased from 66.7% to 74.9%. The proportion reporting home cooking with fresh produce increased from 61.3% to 68.1%. Smaller increases were observed for serving fruits and vegetables between meals and preparing vegetable-based dishes. However, the overall change in the parental practices score was not statistically significant (*p* = 0.770), and no significant between-group differences were observed (*p* = 0.297).

Among participants in the intervention groups, improvement in nutrition-related parenting practices was significantly associated with parent-reported increases in the variety of vegetables consumed by the household at follow-up. Parents who improved their nutrition-related parenting practices had higher odds of reporting an increase in household vegetable variety than those who did not (OR = 2.219, 95% CI 1.057–4.656, *p* = 0.035).

## 4. Discussion

The present study evaluated parental and household dietary outcomes during implementation of the school-based “Leket Health” rescued-produce program. Categorical changes in parent-reported daily fruit servings differed across study groups in the complete-case analysis (*p* = 0.028), whereas FFQ-derived fruit and vegetable intake (g/day) showed no statistically significant between-group differences. No statistically significant between-group difference was observed for changes in parent-reported vegetable servings (*p* = 0.738). Child-reported FFQ-derived fruit and vegetable intake, examined as a secondary exploratory outcome, did not change significantly over the intervention period.

The exploratory finding on parent-reported fruit servings, alongside the null result for FFQ-derived fruit and vegetable intake (g/day), can be considered in light of prior evidence suggesting that fruit consumption may be more amenable to change through behavioral and nutrition interventions than vegetable consumption [24,34,35,36]. Recent evidence further indicates that the effects of interventions on fruit and vegetable intake are often modest, and that increasing vegetable intake may require more targeted, sustained, and multicomponent strategies [37,38,39]. Several factors could contribute to differences in reported fruit and vegetable servings, although these mechanisms were not tested in this study. Fruit is typically consumed raw and requires minimal preparation, whereas vegetables often require washing, chopping, cooking, seasoning, and greater culinary effort. In the present study population, lack of time and effort was identified as a major barrier to vegetable consumption, further supporting this interpretation. Recent studies also suggest that barriers to fruit and vegetable intake differ by food type, with vegetable intake more closely linked to taste preferences, meal routines, cooking skills, and home food availability [40].

The absence of significant changes in child-reported FFQ-derived fruit and vegetable intake should be interpreted cautiously. Although the intervention was implemented through schools and directly reached students in the full intervention group, measurable changes in children’s habitual intake may require longer exposure, greater intervention intensity, or more targeted child-focused behavioral strategies [37,38,39]. Children’s dietary patterns are shaped by multiple interacting factors, including taste preferences, peer norms, school routines, parental practices, and the broader home food environment [37,41,42]. Therefore, changes observed among parents may represent an earlier or more readily measurable stage in the pathway toward household-level dietary change, whereas changes in children’s intake may require a longer follow-up period or more intensive intervention components.

The observed increase in parental fruit consumption is potentially meaningful from a public health perspective. Even modest increases in fruit intake have been associated with reductions in cardiovascular risk, improved metabolic outcomes, and lower mortality rates [2,3]. Therefore, the intervention merits further study with multiple schools per arm to assess behavioral and long-term health outcomes. The discordance between the two intake measures may reflect differences in measurement resolution and reporting biases, including potential FFQ overestimation of fruit and vegetable intake, while substantial variability in FFQ-derived estimates may have limited the precision of estimated changes [43]. An exploratory post-allocation adherence analysis identified an association between the number of produce boxes collected and a parent-reported increase in fruit intake. Each additional box collected was associated with 6.7% higher odds of reporting improved fruit intake (OR = 1.067, 95% CI 1.003–1.135; *p* = 0.040).

Because box collection may reflect parental engagement and self-selection, this association does not establish that collecting more boxes caused the dietary change. The model describes an association with post-allocation adherence and does not estimate an intervention effect. Prior studies of produce prescription and food access interventions suggest that repeated exposure to fresh produce can influence availability and consumption [20,42,44]. The present adherence analysis cannot establish this mechanism.

In the present study, produce-box collection was higher in the full-intervention school than in the partial-intervention school. Differences in collection could also reflect school-specific distribution practices, access, or family engagement. For families in the full intervention group, only 11.5% of invited parents attended the online session. Repeated exposure to educational information sheets and recipes tailored to the produce included in each box was provided; their uptake was not assessed. This practical, box-specific educational component may have supported the use, preparation, and acceptability of the produce provided. However, given the low attendance at the parent session and the unmeasured uptake of printed materials, the full- and partial-arm analyses do not provide a clear comparison between education plus produce and produce alone, and the independent contribution of nutrition education cannot be determined.

Exposure and reinforcement models suggest that repeated access to healthy foods may increase familiarity, acceptance, and eventual incorporation into dietary habits [45,46,47]. The present adherence analysis cannot establish these mechanisms or separate them from parental engagement and self-selection.

An exploratory analysis among participants in the intervention groups identified an association between improved nutrition-related parenting practices and a parent-reported increase in household vegetable variety (OR = 2.219, 95% CI 1.057–4.656; *p* = 0.035). This is consistent with previous research highlighting the central role of parents in shaping the home food environment through food availability, meal structure, preparation practices, and social eating norms [41,42]. However, the overall parenting-practices score showed no statistically significant change (*p* = 0.770) or between-group difference (*p* = 0.297). The OR describes an association between two parent-reported behaviors and does not demonstrate an intervention effect on either outcome.

The findings suggest that while produce provision may improve access and availability, additional behavioral components may be needed to translate access into sustained dietary change, particularly for more complex dietary components such as vegetables [35,37,47].

The study population was characterized by moderate socioeconomic status, with a non-negligible proportion of households experiencing food insecurity. These findings are consistent with previous evidence linking food insecurity to lower dietary quality and reduced fruit and vegetable intake [16,17]. In families with school-aged children, such constraints may influence not only individual intake but also household food availability, purchasing priorities, and meal routines [13].

Household food insecurity increased from 8.7% at baseline to 14.3% at follow-up, worsening across all study arms, with no statistically significant between-group difference in the extent of deterioration (*p* = 0.810). The exploratory interaction analysis cannot establish that the program buffered dietary deterioration. A potential buffering effect remains a hypothesis for future research and was not demonstrated by this study.

Further studies are needed to determine whether food access programs can mitigate dietary deterioration during periods of economic or social instability, when nutritional outcomes may worsen [48,49,50,51].

The magnitude of improvement observed in this study is consistent with prior interventions combining food provision and education. SNAP incentive programs have demonstrated modest increases in fruit and vegetable intake [23], while prescription-based interventions combining access and education have shown larger effects [20]. The present study adds exploratory evidence on parental outcomes within a school-based framework, an area less frequently examined in school-based nutrition interventions [26].

The integration of rescued agricultural produce into a universal school-based program represents an additional contribution. This model links food rescue, sustainability, and nutrition equity, while potentially reducing stigma by offering produce boxes to all families within participating schools rather than targeting only families identified as food insecure. Delivery through schools may further increase reach and acceptability by engaging families through a familiar community setting, particularly in communities where need is present but not always visible through formal welfare systems.

This study has several notable strengths. First, it was conducted in a real-world school setting, enhancing external validity and applicability. Second, it employed a multi-component intervention addressing both food access and behavioral support, which is increasingly recognized as necessary for effective dietary change. Third, the study included a relatively large sample and multiple comparison groups, allowing descriptive comparisons of dietary outcomes across the participating schools. Fourth, the inclusion of parental behavioral measures provides insight into potential household-level pathways of change. In addition, the inclusion of both parent-reported and child-reported data provides a broader view of the school-based intervention context, while preserving the primary analytic focus on parental outcomes. Finally, the study describes implementation and dietary outcomes during a period of national crisis.

Several limitations should be considered. The study used a non-randomized cluster design, with one school assigned to each study arm rather than being randomly allocated. Retention was lower in the control arm than in the intervention arms, raising the possibility that differential attrition may have affected the complete-case findings, although the between-arm retention difference did not reach conventional statistical significance. The allocation-based sensitivity analysis, therefore, included participants with missing data using multiple imputation under a missing-at-random assumption and examined additional assumptions regarding missing follow-up intake data. Although the imputed analysis yielded higher adjusted follow-up fruit intake in both intervention arms than in the control arm, the magnitude of the between-arm differences varied across missing-data scenarios, particularly for the partial-intervention arm. These findings indicate that the direction and magnitude of the observed associations are sensitive to assumptions about missing outcomes and should therefore be interpreted with caution. Moreover, these analyses do not overcome the fundamental design limitation that each study arm comprised a single school; consequently, intervention effects cannot be disentangled from school-level differences, and causal inference is not warranted. Furthermore, associations between box collection and dietary improvement may reflect participant engagement and other confounding factors. Non-randomized allocation and school–intervention confounding further limit causal interpretation. Dietary intake was assessed by self-report, which may be subject to recall and social desirability biases. In addition, significant changes were observed in self-reported servings but not in FFQ-derived intake in grams, suggesting that measurement differences should be considered when interpreting the findings. Child dietary intake was also assessed by self-report using a study-specific FFQ focused on fruits and vegetables. Although the questionnaire included age-appropriate visual aids, reporting accuracy may be limited in this age group. The parent-directed group education component was only partially implemented due to contextual constraints during the study period, and attendance at the online session was limited to 12 of 104 invited parents (11.5%). Therefore, conclusions regarding the independent contribution of parental nutrition education cannot be drawn. The study was conducted in a single geographic area, which may limit its generalizability to other populations or settings. The study period overlapped with the war, which may have affected food access and household food security differently across schools. All within-school pre–post changes, including the deterioration in food security, are therefore interpreted as period-related changes; this design cannot isolate the contribution of the war or the intervention. Finally, the duration of the intervention, while sufficient to detect short-term changes in parental outcomes, may not capture the long-term sustainability of dietary behaviors or the delayed effects on children’s intake.

The findings highlight the need for further evaluation to inform public health practice and policy. School-based interventions that integrate produce provision with nutrition education warrant further study as a potential strategy for improving family-level dietary behaviors, particularly among communities facing socioeconomic constraints. Such programs warrant evaluation to determine whether universal implementation reduces disparities in diet quality, minimizes stigma, and reaches families whose needs may not be captured through formal welfare systems. The integration of food rescue further enhances sustainability and aligns with broader environmental and social goals.

Future studies should examine long-term outcomes, including sustained dietary change, household food environment indicators, and health-related outcomes. Randomized controlled trials would strengthen causal inference. Further research is also needed to examine whether improvements in parental intake, parenting practices, and household produce variety translate into measurable changes in children’s dietary intake over longer follow-up periods. In addition, future interventions should identify strategies specifically targeting vegetable consumption, which remains resistant to change, particularly among children.

## 5. Conclusions

In this study of the “Leket Health” program, parents in the intervention schools more often reported increased fruit servings than parents in the control school. However, no significant between-school differences in changes were observed in parental vegetable servings or FFQ-derived fruit and vegetable intake (g/day) among either parents or children. Parents reported high use of the contents of the produce box, while household food security worsened across all study arms. A potential buffering effect against dietary deterioration remains a hypothesis and was not demonstrated by this study. Because each arm comprised a single school, school-specific and intervention effects cannot be separated. School-based programs that combine rescued produce distribution with nutrition education warrant further evaluation in larger studies with multiple schools per arm, a prespecified primary dietary endpoint, and measures of implementation and acceptability before recommendations for scale-up can be made. Such studies should also examine whether more intensive or sustained educational components are associated with changes in children’s intake and vegetable consumption.

## Figures and Tables

**Table 1:** Baseline demographic and socioeconomic characteristics of participants by study group.

**Parents** **Characteristic**	**Full** **Intervention (** ***n*** **= 80)**	**Partial** **Intervention (** ***n*** **= 66)**	**Control** **(** ***n*** **= 50)**	**Total** **(** ***n*** **= 196)**	***p*** **-Value**
**Age (years)** mean ± SD (range)	41.2 ± 4.8 (31–54)	40.8 ± 5.6 (30–55)	39.8 ± 4.7 (30–53)	40.7 ± 5.1 (30–55)	0.119
**Female** *n* (%)	69 (86.3%)	59 (89.4%)	46 (92.0%)	174 (88.8%)	0.078
**Number of household members** mean ± SD (range)	4.9 ± 1.2 (2–10)	5.0 ± 1.3 (1–10)	4.8 ± 1.0 (3–7)	4.9 ± 1.2 (1–10)	0.704
**Number of household members aged <18 years** mean ± SD (range)	2.7 ± 1.1 (1–6)	2.8 ± 1.2 (1–6)	2.7 ± 1.0 (1–5)	2.7 ± 1.1 (1–6)	0.599
**Country of birth** *n* (%)					
**Israel**	37 (46.3%)	33 (50.0%)	23 (46.0%)	93 (47.4%)	0.330
Eastern Europe *	42 (52.5%)	31 (47.0%)	26 (52.0%)	99 (50.5%)	
Other **	1 (1.3%)	2 (3.0%)	1 (2.0%)	4 (2.1%)	
**Education (years)** mean ± SD (range)	14.7 ± 3.7 (5–25)	14.0 ± 2.9 (5–25)	15.3 ± 3.1 (5–22)	14.6 ± 3.4 (5–25)	0.133
**Unemployed** *n* (%)	4 (5.0%)	6 (9.1%)	1 (2.0%)	11 (5.6%)	0.290
**Food security status** *n* (%)					
**High**	61 (76.3%)	50 (75.8%)	41 (82.0%)	152 (77.6%)	0.129
**Marginal**	16 (20.0%)	10 (15.2%)	1 (2.0%)	27 (13.8%)	
**Low**	2 (2.5%)	5 (7.6%)	8 (16.0%)	15 (7.7%)	
**Very low**	1 (1.3%)	1 (1.5%)	0 (0.0%)	2 (1.0%)	
**Socioeconomic status (SES)** *n* (%)					
**Able to cover monthly household expenses**	55 (70.5%)	42 (64.6%)	33 (66.0%)	130 (67.4%)	0.072
**Income above the national average**	47 (60.3%)	31 (47.7%)	28 (56.0%)	106 (54.9%)	0.293
**Income similar to the national average**	17 (21.8%)	17 (26.2%)	17 (34.0%)	51 (26.4%)	
**Income below the national average**	7 (8.8%)	10 (15.4%)	2 (4.0%)	19 (9.8%)	
**Unknown**/**Refused**	7 (9.0%)	7 (10.8%)	3 (6.0%)	17 (8.8%)	
**Children** **Characteristic**	**Full** **Intervention (** ***n*** **= 101)**	**Partial** **Intervention (** ***n*** **= 79)**	**Control** **(** ***n*** **= 73)**	**Total** **(** ***n*** **= 253)**	***p*** **-Value**
**Boys** *n* (%)	48 (47.5%)	48 (60.8%)	35 (47.9%)	131 (51.8%)	0.156
**Grade** *n* (%)					
**Grade 4**	53 (52.5%)	42 (53.2%)	29 (39.7%)	124 (49.0%)	0.170
**Grade 5**	48 (47.5%)	37 (46.8%)	44 (60.3%)	129 (51.0%)	
**Country of birth** *n* (%)					
**Israel**	93 (92.1%)	77 (97.5%)	71 (97.3%)	241 (95.3%)	0.153
Other ***	8 (7.9%)	2 (2.5%)	2 (2.7%)	12 (4.7%)	

Values are presented as mean ± standard deviation (SD) for continuous variables and *n* (%) for categorical variables. Differences between groups were assessed using one-way ANOVA for normally distributed variables, Kruskal–Wallis test for non-normally distributed variables, and chi-square test for categorical variables. SES = socioeconomic status. A *p*-value < 0.05 was considered statistically significant. * Eastern Europe = Uzbekistan, Ukraine, Azerbaijan, Belarus, Georgia, Tajikistan, Latvia, Lithuania, Kazakhstan, Kyrgyzstan, Russia. ** Other = United States, Ethiopia, France. *** Other = Ukraine, Germany, Kyrgyzstan, Russia.

**Table 2:** Changes in fruit and vegetable consumption following the intervention.

Characteristic	Full Intervention (*n* = 80)	Partial Intervention (*n* = 66)	Control (*n* = 50)	Total	*p*-Value
**Change in vegetable consumption, ** ***n*** **(%)**	**(** ***n*** **= 71)**	**(** ***n*** **= 63)**	**(** ***n*** **= 44)**	**(** ***n*** **= 178)**	0.738
Decreased intake	24 (33.8%)	21 (33.3%)	18 (40.9%)	63 (35.4%)	
No change in intake	26 (36.6%)	25 (39.7%)	13 (29.5%)	64 (36.0%)	
Increased intake	21 (29.6%)	17 (27.0%)	13 (29.5%)	51 (28.7%)	
**Change in fruit** **consumption, ** ***n*** **(%)**	**(** ***n*** **= 75)**	**(** ***n*** **= 64)**	**(** ***n*** **= 42)**	**(** ***n*** **= 181)**	0.028 *
Decreased intake	17 (22.7%)	23 (35.9%)	17 (40.5%)	57 (31.5%)	
No change in intake	37 (49.3%)	20 (31.3%)	19 (45.2%)	76 (42.0%)	
Increased intake	21 (28.0%)	21 (32.8%)	6 (14.3%)	48 (26.5%)	

Changes are presented as categorical changes from baseline to post-intervention based on self-reported daily servings: decreased intake, no change, or increased intake. Between-group comparisons were conducted using chi-square tests. * *p*-value < 0.05 was considered statistically significant. Outcome-specific denominators and confirming that percentages were calculated using the corresponding denominators within each study arm.

**Table 3:** Allocation-based sensitivity analyses of parent-reported fruit servings/day.

Missing Data Scenario	Full Intervention vs. Control	Partial Intervention vs. Control
Multiple Imputation under MAR	+0.38 (0.04 to 0.71)	+0.25 (−0.1 to 0.61)
No change, missing follow-up = baseline	+0.29 (0.02 to 0.56)	+0.27 (−0.02 to 0.56)
Adverse missingness	+0.15 (−0.20 to 0.50)	0.06 (−0.30 to 0.43)

Values are adjusted differences versus the control, in portions/day, with nominal 95% confidence intervals. Analyses retained 269 identifiable participants according to original school-level allocation and adjusted for baseline fruit intake, parental age, education, and baseline household income category. MAR analyses used 100 imputations. In the no-change scenario, missing follow-up values were set equal to observed or imputed baseline values. In the adverse scenario, one portion/day was subtracted from imputed follow-up values for intervention participants with missing outcomes, with a minimum of one portion/day. With one school per arm, intervals do not account for school-level clustering.

**Table 4:** Post-allocation logistic regression analysis of the association between produce-box collection and improved parental fruit consumption.

Variable	OR	95% CI	*p*-Value
Number of boxes collected	1.067	1.003–1.135	0.040 *
Socioeconomic status: able to cover household expenses	0.512	0.216–1.214	0.129
Constant	0.222	-	<0.001 *

An exploratory post-allocation multivariable logistic regression model examining the association between the number of produce boxes collected and improved parental fruit intake. Improved fruit intake was defined as an increase in self-reported daily fruit servings from baseline to follow-up. The model was adjusted for the reported ability to cover monthly household expenses. Results are presented as odds ratios (ORs) with 95% confidence intervals (CIs). The analysis was based on participants with complete data for the outcome and model covariates (*n* = 181). Because box collection occurred after study allocation, this model represents an adherence/exposure analysis rather than an analysis by original study allocation. * *p* < 0.05 was considered statistically significant.

## Data Availability

The data presented in this study are available on request from the corresponding author. The data are not publicly available due to privacy and ethical restrictions.

## Supplementary Materials

The following supporting information can be downloaded at: https://www.mdpi.com/article/10.3390/nu18203355/s1, Table S1: Manuscript location and clarification; Table S2: Allocation-based analyses of parent-reported fruit intake under alternative assumptions for missing data.

## Abbreviations

The following abbreviations are used in this manuscript:

ANOVA	Analysis of variance
APC	Article processing charge
CI	Confidence interval
FFQ	Food frequency questionnaire
OR	Odds ratio
SD	Standard deviation
SES	Socioeconomic status
SPSS	Statistical Package for the Social Sciences
USDA	United States Department of Agriculture

## References

[1] World Health Organization (2003). Diet, Nutrition and the Prevention of Chronic Diseases: Report of a Joint WHO/FAO Expert Consultation.

[2] Wang X., Ouyang Y., Liu J., Zhu M., Zhao G., Bao W., Hu F.B. (2014). Fruit and Vegetable Consumption and Mortality from All Causes, Cardiovascular Disease, and Cancer: Systematic Review and Dose-Response Meta-Analysis of Prospective Cohort Studies. BMJ.

[3] Aune D., Giovannucci E., Boffetta P., Fadnes L.T., Keum N., Norat T., Greenwood D.C., Riboli E., Vatten L.J., Tonstad S. (2017). Fruit and Vegetable Intake and the Risk of Cardiovascular Disease, Total Cancer and All-Cause Mortality—A Systematic Review and Dose-Response Meta-Analysis of Prospective Studies. Int. J. Epidemiol..

[4] Slavin J.L., Lloyd B. (2012). Health Benefits of Fruits and Vegetables. Adv. Nutr..

[5] Israeli Ministry of Health Israel’s New National Nutrition Recommendations—The New Nutritional Rainbow. https://www.gov.il/en/pages/dietary-guidelines.

[6] Shahar D., Shai I., Vardi H., Shahar A., Fraser D. (2005). Diet and Eating Habits in High and Low Socioeconomic Groups. Nutrition.

[7] Israel Center for Disease Control (ICDC) Knowledge, Attitudes, Practices (KAP) Survey 2023–2024: Selected Findings. https://www.gov.il/he/pages/kap2023-2024.

[8] Israel Center for Disease Control (ICDC) The Fourth Israel National Health Interview Survey INHIS-4. https://www.gov.il/en/pages/02082022-02.

[9] OECD Health Statistics Consumption of Vegetables and Fruits, Daily—Survey Data. https://webfs-els.oecd.org/files/Health-Statistics/HEALTH_LVNG_Risk-Factors-for-Health/4_Food-supply-and-consumption.pdf.

[10] Social Survey 2017: Dietary Habits. https://www.cbs.gov.il/he/publications/DocLib/2019/seker_hevrati17_1761/h_print.pdf.

[11] Social Survey 2023: Health and Nutrition Behaviors. https://www.cbs.gov.il/he/mediarelease/DocLib/2024/281/19_24_281b.pdf.

[12] Durward C.M., Savoie-Roskos M., Atoloye A., Isabella P., Jewkes M.D., Ralls B., Riggs K., LeBlanc H. (2019). Double Up Food Bucks Participation Is Associated with Increased Fruit and Vegetable Consumption and Food Security Among Low-Income Adults. J. Nutr. Educ. Behav..

[13] Eicher-Miller H.A., Graves L., McGowan B., Mayfield B.J., Connolly B.A., Stevens W., Abbott A. (2023). A Scoping Review of Household Factors Contributing to Dietary Quality and Food Security in Low-Income Households with School-Age Children in the United States. Adv. Nutr..

[14] FAO An Introduction to the Basic Concepts of Food Security. https://www.fao.org/4/al936e/al936e00.pdf.

[15] Rome Declaration on World Food Security. https://www.fao.org/3/w3613e/w3613e00.htm.

[16] Araújo M.L.D., Mendonça R.D.D., Lopes Filho J.D., Lopes A.C.S. (2018). Association between Food Insecurity and Food Intake. Nutrition.

[17] Turnbull O., Homer M., Ensaff H. (2021). Food Insecurity: Its Prevalence and Relationship to Fruit and Vegetable Consumption. J. Hum. Nutr. Diet..

[18] Rico-Campà A., Martínez-González M.A., Alvarez-Alvarez I., Mendonça R.D.D., De La Fuente-Arrillaga C., Gómez-Donoso C., Bes-Rastrollo M. (2019). Association between Consumption of Ultra-Processed Foods and All Cause Mortality: SUN Prospective Cohort Study. BMJ.

[19] Aramburu A., Alvarado-Gamarra G., Cornejo R., Curi-Quinto K., Díaz-Parra C.D.P., Rojas-Limache G., Lanata C.F. (2024). Ultra-Processed Foods Consumption and Health-Related Outcomes: A Systematic Review of Randomized Controlled Trials. Front. Nutr..

[20] Slagel N., Newman T., Sanville L., Dallas J., Cotto-Rivera E., Moore J., Roberts Mph A., Sun Lee J. (2022). Effects of a Fruit and Vegetable Prescription Program with Expanded Education for Low-Income Adults. Health Educ. Behav..

[21] Koyratty N., Azupogo F., Smith T.J., Hinnouho G.-M., Tharaney M., Bliznashka L., Olney D.K., Hess S.Y. (2025). Impact of Intervention Strategies on Fruit and Vegetable Intake in Low-, Middle- and High-Income Countries: A Scoping Review. Food Nutr. Bull..

[22] Lock K., Pomerleau J., Knai C., McKee M. (2006). Improving the Worldwide Evidence-Based Effective Interventions and Programmes Designed to Increase Fruit and Vegetable Intake. Rev. Chil. Nutr..

[23] Karpyn A., Pon J., Grajeda S.B., Wang R., Merritt K.E., Tracy T., May H., Sawyer-Morris G., Halverson M.M., Hunt A. (2022). Understanding Impacts of SNAP Fruit and Vegetable Incentive Program at Farmers’ Markets: Findings from a 13 State RCT. Int. J. Environ. Res. Public. Health.

[24] Sharma S.V., Markham C., Chow J., Ranjit N., Pomeroy M., Raber M. (2016). Evaluating a School-Based Fruit and Vegetable Co-Op in Low-Income Children: A Quasi-Experimental Study. Prev. Med..

[25] Prelip M., Le Thai C., Toller Erausquin J., Slusser W. (2011). Improving Low-income Parents’ Fruit and Vegetable Intake and Their Potential to Impact Children’s Nutrition. Health Educ..

[26] Abderbwih E., Mahanani M.R., Deckert A., Antia K., Agbaria N., Dambach P., Kohler S., Horstick O., Winkler V., Wendt A.S. (2022). The Impact of School-Based Nutrition Interventions on Parents and Other Family Members: A Systematic Literature Review. Nutrients.

[27] Israel Center for Disease Control (ICDC) Rav Mabat Adult Second National Health and Nutrition Survey Ages 18–64 2014–2016. https://www.gov.il/he/pages/mabat-adults-2014-2016-383.

[28] Central Bureau of Statistics (CBS) Household Income and Expenditure—Data from the 2019 Survey. https://www.cbs.gov.il/he/publications/doclib/2022/1869/t01_01.pdf.

[29] Economic Research Service U.S. Household Food Security Survey Module: Three-Stage Design, with Screeners. https://www.ers.usda.gov/media/6422/us-household-food-security-survey-module.pdf.

[30] Shai I., Rosner B.A., Shahar D.R., Vardi H., Azrad A.B., Kanfi A., Schwarzfuchs D., Fraser D. (2005). Dietary Evaluation and Attenuation of Relative Risk: Multiple Comparisons between Blood and Urinary Biomarkers, Food Frequency, and 24-Hour Recall Questionnaires: The DEARR Study. J. Nutr..

[31] Israel Ministry of Health Food Groups and Portion Sizes. https://www.gov.il/he/pages/food-dish-size.

[32] Kaufman-Shriqui V., Entin-Kreizman A., Fraser D., Novack Y., Bilenko N., Vardi H., Elhadad N., Shahar D.R. (2013). Development and Validation of a Food Frequency Questionnaire for Preschool Children Using Multiple Methods. Int. J. Child Health Nutr..

[33] Campbell M.K., Piaggio G., Elbourne D.R., Altman D.G., CONSORT Group (2012). CONSORT 2010 statement: Extension to cluster randomised trials. BMJ.

[34] Dave J.M., Evans A.E., Pfeiffer K.A., Watkins K.W., Saunders R.P. (2010). Correlates of Availability and Accessibility of Fruits and Vegetables in Homes of Low-Income Hispanic Families. Health Educ. Res..

[35] Wolfenden L., Barnes C., Lane C., McCrabb S., Brown H.M., Gerritsen S., Barquera S., Véjar L.S., Munguía A., Yoong S.L. (2021). Consolidating Evidence on the Effectiveness of Interventions Promoting Fruit and Vegetable Consumption: An Umbrella Review. Int. J. Behav. Nutr. Phys. Act..

[36] Øvrum A., Bere E. (2014). Evaluating Free School Fruit: Results from a Natural Experiment in Norway with Representative Data. Public Health Nutr..

[37] Hendrie G.A., Anastasiou K., Brindal E., Wiggins B., Baird D.L., Johnson B.J., Bell L.K., Gardner C., Arguelles J.C., Kelaart A. (2024). Increasing Children’s Vegetable Consumption: Translating a Review of the Evidence Base to Develop Best Practice Guidelines. AJPM Focus.

[38] Hodder R.K., O’Brien K.M., Wyse R.J., Tzelepis F., Yoong S., Stacey F.G., Wolfenden L. (2024). Interventions for Increasing Fruit and Vegetable Consumption in Children Aged Five Years and Under. Cochrane Database Syst. Rev..

[39] Froome H., Cheung K.L., Martin W., Norris E. (2024). Identifying and Characterising Digital Behaviour Change Interventions to Improve Fruit and Vegetable Intake in Low-Socioeconomic Status Primary School Children: A Systematic Review. BMC Digit. Health.

[40] Baraldi L.G., Grilo M.F., Landert M., Martins C.A. (2025). Beyond the Family’s Cooking Frequency: The Influence of Cooking Techniques on Vegetable and Fruit Consumption among the U.S. Population. Appetite.

[41] Trofholz A.C., Tate A.D., Draxten M.L., Neumark-Sztainer D., Berge J.M. (2016). Home Food Environment Factors Associated with the Presence of Fruit and Vegetables at Dinner: A Direct Observational Study. Appetite.

[42] Marshall A.N., Markham C., Ranjit N., Bounds G., Chow J., Sharma S.V. (2020). Long-Term Impact of a School-Based Nutrition Intervention on Home Nutrition Environment and Family Fruit and Vegetable Intake: A Two-Year Follow-up Study. Prev. Med. Rep..

[43] El Mesmoudi N., Al Dhaheri A.S., Feehan J., Stojanovska L., Ali H.I. (2022). Validation of a quantitative web-based food frequency questionnaire to assess dietary intake in the adult Emirati population. PLoS ONE.

[44] Bhat S., Coyle D.H., Trieu K., Neal B., Mozaffarian D., Marklund M., Wu J.H.Y. (2021). Healthy Food Prescription Programs and Their Impact on Dietary Behavior and Cardiometabolic Risk Factors: A Systematic Review and Meta-Analysis. Adv. Nutr..

[45] Ling J., Zahry N.R., Robbins L.B. (2019). Dose-Response Relationship in a Healthy Habits Study for Head Start Preschoolers. Nurs. Res..

[46] Lippke S., Corbet J.M., Lange D., Parschau L., Schwarzer R. (2016). Intervention Engagement Moderates the Dose–Response Relationships in a Dietary Intervention. Dose-Response.

[47] Appleton K.M., Hemingway A., Rajska J., Hartwell H. (2018). Repeated Exposure and Conditioning Strategies for Increasing Vegetable Liking and Intake: Systematic Review and Meta-Analyses of the Published Literature. Am. J. Clin. Nutr..

[48] Dufy C. (2023). Food Security in Times of War: Double Discourse and Violent Conflicts. The Case of Russia, 2014–2022. Int. Sociol..

[49] Brinkman H.-J., De Pee S., Sanogo I., Subran L., Bloem M.W. (2010). High Food Prices and the Global Financial Crisis Have Reduced Access to Nutritious Food and Worsened Nutritional Status and Health. J. Nutr..

[50] Fiandrino S., Dowd C., Martini G., Mejova Y., Omodei E., Paolotti D., Tizzani M. (2023). Impact of Food-Related Conflicts on Self-Reported Food Insecurity. Front. Sustain. Food Syst..

[51] Kryshtanovych M., Cherkaska V., Kushnir Y., Buletsa N., Herzanych V. (2023). The impact of the war on food security in Ukraine in the current conditions of socio-economic development. Cuest. Políticas.

