# Supplementary material for: School-Based Rescued Produce Distribution, and Changes in Parental Fruit Intake and Household Dietary Behaviors: A Non-Randomized Controlled Cluster Trial
Source: Nutrients. 2026 Oct 13;18(20):3355. doi: 10.3390/nu18203355 (PMC13708734; doi:10.3390/nu18203355)
Supplement: Supplementary file 1 [file nutrients-18-03355-s001.zip › nutrients-4571565-supplementary.pdf]

Supplementary Table S1: Manuscript location and clarification

**Reporting item**	**Manuscript location and clarification**
Design and cluster structure	Title, Abstract, §2.1: non-randomized cluster-controlled trial; three schools, one per arm
Eligibility and allocation	§§2.2–2.3: school and participant eligibility; principals' willingness; non-random allocation
Intervention components	§2.3: produce distribution and nutrition education
Outcomes and measurements	§2.4: parental servings and FFQ; child FFQ labeled exploratory
Sample-size calculation and analysis	§§2.5–2.6: individual-level calculation; clustering limitations; outcome-specific complete cases
Participant flow and denominators	Figure 1, §3.1, Table 2, and footnote
Interpretation and limitations	Discussion: school–intervention confounding and potentially anti-conservative confidence intervals
Ethics and registration	§2.7 and declarations: approval chronology and confirmed registration status

Supplementary Table S2: Allocation-based analyses of parent-reported fruit intake under alternative assumptions for missing data

Missing-data analysis	Full intervention	Partial intervention	Control	Full vs control, adjusted difference (95% CI)	Partial vs control, adjusted difference (95% CI)
**Multiple imputation under MAR**	2.81 (2.58–3.04)	2.69 (2.45–2.92)	2.44 (2.18–2.69)	**+0.38 (0.04 to 0.71)**	**+0.25 (−0.10 to 0.61)**
**Missing follow-up = baseline**	—	—	—	**+0.29 (0.02 to 0.56)**	**+0.27 (−0.02 to 0.56)**
**Adverse missing-outcome scenario**	—	—	—	**+0.15 (−0.20 to 0.50)**	**+0.06 (−0.30 to 0.43)**

Values for the multiple-imputation analysis are adjusted follow-up means in portions/day with nominal 95% confidence intervals. Missing outcome and covariate data were addressed using 100 multiple imputations under a missing-at-random (MAR) assumption. Models were adjusted for baseline fruit intake, parental age, education, and baseline household income category. In the no-change scenario, missing follow-up fruit intake was set equal to baseline intake. In the adverse missing-outcome scenario, one portion/day was subtracted from imputed follow-up values among intervention participants with missing follow-up outcomes, with a minimum value of one portion/day. All analyses retained participants according to their original school-level allocation. Because each study arm comprised a single school, confidence intervals are nominal individual-level estimates and do not account for clustering; the analyses cannot distinguish intervention effects from school-level differences.
